# Diaphragmatic Palsy Due to a Paraneoplastic Autoimmune Syndrome Revealed by Checkpoint Inhibitors

**DOI:** 10.3390/reports7040084

**Published:** 2024-10-11

**Authors:** Jean-Baptiste Destival, Jean-Marie Michot, Cécile Cauquil, Nicolas Noël, Salima Hacein-Bey-Abina, Pascale Chrétien, Olivier Lambotte

**Affiliations:** 1 Service de Médecine Interne et Immunologie Clinique, Hôpital Bicêtre, Assistance Publique—Hôpitaux de Paris, Groupe Hospitalier Universitaire Paris Saclay, 94270 Le Kremlin-Bicêtre, Franceolivier.lambotte@aphp.fr (O.L.); 2 Département d’Innovation Thérapeutique et des Essais Précoces (DITEP), Institut de Cancérologie Gustave Roussy, 94800 Villejuif, France; jean-marie.michot@gustaveroussy.fr; 3 Assistance Publique—Hôpitaux de Paris, Service de Neurologie, Groupe Hospitalier Universitaire Paris Saclay, Hôpital Bicêtre, 94270 Le Kremlin-Bicêtre, France; cecile.cauquil@aphp.fr; 4 Faculté de médecine, Université Paris Saclay, National Institute of Health and Medical Research (INSERM) UMR-1184, Atomic Energy and Alternative Energies Commission (CEA), 94270 Le Kremlin Bicêtre, France; 5 Assistance Publique—Hôpitaux de Paris, Service d’Immunologie Biologique, Groupe Hospitalier Universitaire Paris Saclay, Hôpital Bicêtre, 94270 Le Kremlin-Bicêtre, France; salima.hacein-bey@aphp.fr (S.H.-B.-A.); pascale.chretien@aphp.fr (P.C.); 6 Unit of Chemical and Biological Technologies for Health (UTCBS), National Center for Scientific Research (CNRS) UMR-8258, INSERM U1267, Faculté de Pharmacie de Paris, Université Paris Cité, 75006 Paris, France

**Keywords:** respiratory paralysis, paraneoplastic syndromes, immune checkpoint inhibitors, myasthenia gravis, myositis

## Abstract

**Background and Clinical Significance:** Immune checkpoint inhibitors (ICIs) have revolutionized cancer treatment but may underlie diverse and potentially life-threatening immune-related adverse events (irAEs). They may cause various conditions leading to respiratory failure, including myasthenic syndromes and myositis. However, diaphragmatic paralysis (DP) has rarely been reported. To describe patients with diaphragmatic paralysis in a pharmacovigilance registry, we searched the prospective REISAMIC registry at the Gustave Roussy Cancer Center (Villejuif, France) for cases of diaphragmatic palsy (DP) occurring from September 2014 to December 2021. **Case Presentation:** We identified three patients, in whom DP was confirmed by diaphragmatic ultrasonography, pulmonary function tests, and/or diaphragmatic electroneuromyogram. Diaphragmatic palsy was life-threatening in all patients, as it caused respiratory failure requiring mechanical ventilation. In all cases, a pre-existing subclinical paraneoplastic syndrome was detected. Onconeural antibodies (anti-titin and anti-VGCC) were detected in these patients before and after the initiation of ICI therapy, suggesting a mixed paraneoplastic syndrome with features overlapping those of myasthenic syndrome (myasthenia gravis in one patient and Lambert–Eaton syndrome in another) and myositis. **Conclusions:** Diaphragmatic palsy is a severe irAE potentially resulting from different mechanisms, including myositis and neuromuscular junction involvement (myasthenia gravis, Lambert–Eaton). Antineuronal antibodies associated with such conditions were already present in our patients prior to immunotherapy initiation, suggesting ICIs could trigger flare-ups of pre-existing silent paraneoplastic autoimmune conditions.

## 1. Introduction and Clinical Significance

Immune checkpoint inhibitors (ICI) restore the immune response directed against cancer cells, but they also frequently cause immune-related adverse events (irAEs) and increase the risk of flare-ups of pre-existing autoimmune diseases or paraneoplastic syndromes (PNS) [1].

Life-threatening irAEs are rare and can present as myositis and myocarditis overlapping with neurologic syndromes such as myasthenia gravis [2]. Patients with myasthenia-like syndromes induced by ICIs present with muscle weakness, oculomotor disorders, and ptosis, but tests for autoantibodies are generally negative [3]. Respiratory failure may occur due to pneumonitis, but also due to respiratory muscle dysfunction caused by neuritis, myasthenic syndromes or myositis. The diaphragm plays a major role in these conditions, but diaphragmatic palsy (DP) has rarely been reported [4].

We report here three cases of DP in the setting of paraneoplastic myasthenia-like syndromes revealed by the initiation of treatment with ICIs.

## 2. Case Presentation

### 2.1. Patients

We searched the prospective academic *Registre des Effets Indésirables Sévères des Anticorps Monoclonaux Immunomodulateurs en Cancérologie* (REISAMIC) registry for cases of DP occurring from September 2014 to December 2021. Since September 2014, the REISAMIC registry has prospectively collected all irAEs of grade 2 or above occurring in adult patients treated with ICIs in real-life conditions at the Gustave Roussy Cancer Center (Villejuif, France). Each irAE was recorded in the patient’s medical file by the patient’s physician and was included in the study by the registry’s pharmacovigilance team.

### 2.2. Autoantibodies

The patients were tested for onconeural antibodies by the immunodot method (Ravo®, Freiburg, Germany) at the immunology laboratory of Bicêtre Hospital. All patients were tested for anti-Yo, anti-Hu, anti-RI, anti-amphyphysin, anti-CV2, anti-Ma1, anti-Ma2, anti-GAD, anti-Sox1, anti-Tr, anti-Zic4, anti-recoverin, anti-titin, and anti-PKC gamma antibodies.

Tests were performed for anti-voltage-gated calcium channel (VGCC) antibodies in patient #3, by both an independent laboratory using RiaRSR® (Cardiff, UK) VGCC antibody and in a specialized CNRS research laboratory in Marseille, France.

### 2.3. Results

Three patients with DP were identified among the 2610 patients included in the REISAMIC registry during the study period (prevalence of 0.4% among irAEs of grade 2 and above). Their characteristics are presented in Table 1. All were treated with PD-1 inhibitors for a metastatic cancer and were hospitalized in the ICU for respiratory failure. The respiratory symptoms occurred within two to six weeks of the initiation of ICI treatment. Chest CT scans were normal in two patients and showed pneumonia in the third, retrospectively attributed to anti-PD-1 treatment. Arterial blood gas analyses revealed moderate hypercapnia patients #1 and #2 (56 and 66 mmHg, respectively). The respiratory symptoms rapidly worsened, leading to mechanical ventilation in two patients and noninvasive ventilation in the remaining patient.

The patients also displayed myalgia and global muscle weakness (all patients), including swallowing disorders, binocular diplopia (patients #2 and #3), dysarthria (patient #3) asymmetric ptosis and drooping of the head (patient #2).

DP was suspected due to the muscle symptoms and difficulties observed during weaning off ventilation, and more investigations were performed. Pulmonary function tests (PFT) revealed a low forced vital capacity (FVC) in all patients. Maximal inspiratory pressure was measured in patient #3 and was also found to be low. Ultrasound scans of the diaphragm were performed in patients #1 and #2 and showed complete DP (less than 20% of diaphragmatic excursion compared to a healthy patient for patient #1, and complete DP without precision in patient #2). Electromyograms (EMG) were obtained from all patients and showed signs of myogenic involvement in patients #1 and #2, and signs of axonal neuropathy in patients #1 and #3. A diaphragmatic electromyogram (Supplementary S1) was performed only in patient #3 and revealed a very low compound muscle action potential (CMAP) on both left and right diaphragms and a moderately low CMAP amplitude on the right spinal and facial nerves. No increment or decrement was observed after repeated stimulation and maximal effort for 30 s, respectively. The final diagnosis was DP in all three patients.

Blood tests initially revealed abnormally high levels of muscle enzymes (creatine phosphokinases) in patients #1 and #2. Muscle MRI revealed multifocal muscle edema consistent with myositis in all patients (confirmed by muscle biopsy in one patient). High levels of cardiac enzymes were also detected in patients #1 and #2, and myocarditis was confirmed by cardiac MRI in patient #1. Cerebrospinal fluid examination was performed in patients #1 and #3 and yielded normal results. Spinal MRI was performed on patient #1 and revealed multifocal inflammatory lesions, especially at the C2 and C3 levels, suggesting associated myelitis. C-reactive protein (CRP) was elevated in patient #1 (34 mg/L) and patient #2 (31 mg/L) and was negative in patient #3.

**Figure 1:**
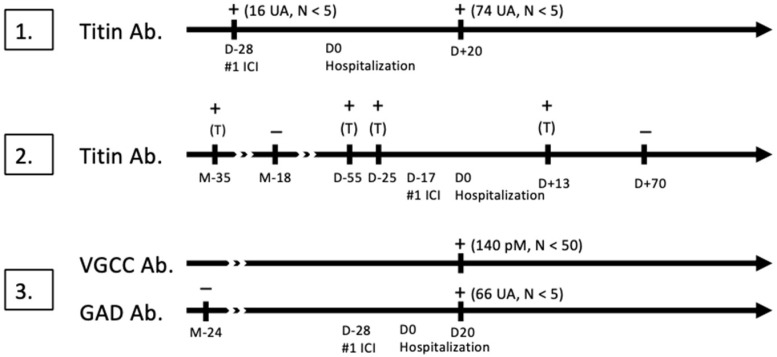
Antibody testing in each patient, before and after the first administration of immune-checkpoint inhibitor (#1 ICI). D0 Hospitalization marks the day of admission for respiratory failure. + indicates a positive test, − indicates a negative test. Antibody titers are indicated in UA (antibody unit) or pM (pmmol/L). T means trace of antibody. D: day, M: month, VGCC: voltage-gated calcium channel, Ab: antibody.

Serum samples obtained from the patients were tested for autoantibodies implicated in PNS. Tests for onconeural antibodies were performed in all patients and were positive for anti-titin antibodies in patients #1 (16 antibody units, AU) and #2 (4 AU), and for anti-GAD (66 AU) antibodies in patient #3. Positive results were also obtained in tests for anti-voltage-gated calcium channel (VGCC) antibodies in patient #3 (140 pM, N < 50). Blood samples collected before the initiation of immunotherapy were tested retrospectively and were found to contain the same antibodies against titin and GAD detected after treatment initiation. However, it was technically not possible to retrospectively test the blood sample of patient #3 for anti-VGCC antibodies due to a too long duration of conservation. The antibody testing results are presented in Figure 1.

Anti-PD-1 therapy was stopped in all three patients. Two patients (#1 and #3) were treated with amifampridine (a VGCC agonist) and pyridostigmine (an acetylcholinesterase inhibitor). All patients received high-dose corticosteroid therapy, and patients #1 and #2 received intravenous immunoglobulins (IVIg). JAK inhibitors were tested in two patients (#1 and #2), with limited success. Infliximab treatment failed in patient #3. Only tacrolimus, used in patient #1 as a third line of treatment, significantly improved respiratory symptoms, making it possible to remove the tracheostomy cannula two months after ICU admission. Patient #2 was able to leave the ICU but died from pneumonitis. Patient #3 died in the ICU from tracheostomy obstruction.

The anti-tumoral efficacy of immunotherapy was re-evaluated in all three patients: the cancer had stabilized in patients #1 and #2 but progressed in patient #3.

Anti-titin antibodies were detected by the immunodot method (Ravo). Anti-GAD antibodies were detected by the immunodot method, with confirmation by indirect immunofluorescence assay on primate cerebellum. Anti-VGCC antibodies (P/Q subcategory) were detected with an antibody assay.

## 3. Discussion

We report here three cases of DP triggered by anti-PD-1 treatment. In all three cases, the patients tested positive for onconeural autoantibodies (against titin in two patients and against VGCC in the remaining patient) and had critical disease, leading to death in two cases. Retrospective testing showed that positivity for anti-titin antibodies preceded the first administration of ICIs. These findings suggest that the patients had a pre-existing subclinical PNS, which was asymptomatic before treatment and unmasked by ICIs.

DP occurred unusually rapidly for a PD-1 inhibitor-mediated irAE, providing additional support for this hypothesis. We previously showed that up to half of the patients with known PNS present a worsening symptoms after the initiation of immunotherapy [1]. The existence of an underlying autoimmune mechanism before the administration of ICI therapy is particularly well documented in diabetes. Indeed, patients with cancer who developed autoimmune diabetes mellitus after anti-PD1 therapy have been found to have diabetes-related autoantibodies before treatment [5].

Diaphragmatic dysfunction may have diverse, even multifactorial causes in patients treated with ICIs. A recent communication presented three cases of phrenic nerve palsies after initiation of ICI; however, the only documented mechanism was myositis, and no immunological test was performed [6]. Myasthenia-like syndromes are now well-documented among the irAEs [7], including Lambert–Eaton myasthenic syndrome. Patients with this PNS present with muscle weakness that may be suggestive of myopathy, dysautonomia, involvement of the cranial nerves, and finally respiratory involvement. It is frequently associated with small-cell lung cancers, and anti-VGCC autoantibodies can be detected in two thirds of cases. There have been reports of cases of the worsening or onset of Lambert–Eaton syndrome after the initiation of ICI treatment [8,9]. Anti-titin autoantibodies are frequently found in myasthenia gravis, in association with RACh Ab, mostly in a context of thymic abnormalities. However, these antibodies may also be associated with MuSK and LRP4 antibodies. Myasthenia gravis with anti-titin Abs only seems to be more frequently responsible for respiratory involvement and is less responsive to conventional therapy [10]. This antibody profile may be prevalent in patients with myasthenia gravis and myositis/myocarditis overlap syndrome [9], the main presentation in patients #1 and #2.

Nevertheless, these antibodies lack specificity, as they can be found in many PNS [11], and their presence in isolation cannot be considered to indicate that the patient has myasthenia gravis.

EMG did not confirm neuromuscular junction involvement in any of the three patients. This lack of confirmation is frequent and can be explained principally by technical difficulties, especially when performed in the ICU. The examination should include stimulation-detection, detection, and checking for an increase or decrease. The axonal neuropathy findings observed on the diaphragmatic EMG of patient #3 are classically observed in Lambert–Eaton disease.

Anti-GAD antibodies were found to be present at consistently high levels in the third patient. These antibodies are known to cause stiff-person syndrome and limbic encephalitis. They can also sometimes be responsible for diaphragmatic involvement.

In addition to myasthenia-like syndromes, muscle dysfunction due to myositis plays a role in diaphragmatic palsy. Muscle involvement can sometimes be difficult to highlight on EMG, but this should be seen in perspective with the focal involvement of myositis [2]. It has been shown that false-negative results can be obtained on EMG in cases of myositis induced by immunotherapy if the examination is not performed on the affected area. Muscle MRI examinations can make a useful contribution in this context [12].

Therapeutically, it is difficult to draw conclusions about the individual effectiveness of each of the treatments administered. In patient #1, the introduction of tacrolimus treatment seems to have been decisive for both cardiac and respiratory involvement. The potential value of tacrolimus is supported by other published cases of successful treatment [13]. The American recommendations are based on the cessation of ICI treatment, strong corticosteroid therapy, IV-Ig, plasma exchanges, and the use of another immunosuppressant [14]. In their recent report on 40 patients, Salem et al. suggested the use of abatacept and ruxolutinib [15]. This management regimen can be complemented with specific treatments, such as pyridostigmine or amifampridine, for myasthenia gravis and Lambert–Eaton syndrome, respectively.

## 4. Conclusions

In practice, particular care should be taken when treating patients with DP, because DP may be a manifestation of a pre-existing PNS boosted by ICIs. Rapid diagnosis assisted by clinical findings, CPK and immunological tests, PFT, diaphragmatic ultrasonography, and electrophysiological tests is, therefore, essential to optimize treatment.

## Figures and Tables

**Table 1:** Patient characteristics.

Patient Characteristics			
Patient No.	1	2	3
Cancer type	Metastatic bronchial adenocarcinoma	Metastatic tongue squamous cell carcinoma	Metastatic invasive ductal carcinoma of the breast
Duration (years)	1.5 years	3 years	22 years
Previous lines of therapy (*n*)	1	2	3
ICIs	Nivolumab	Nivolumab	Pembrolizumab
**Diagnosis of diaphragmatic dysfunction**			
Time from ICI initiation to first symptoms (days)	15	17	57
FVC (%)	14%	32%	17%
Maximal inspiratory pressure (MIP)	-	-	6 cmH20
Diaphragmatic ultrasound	Complete paralysis	Complete paralysis	Not performed
Diaphragmatic EMG	Not performed	Not performed	Abnormal
**Etiological diagnosis**			
Myositis	Confirmed	Confirmed	Probable
*CPK (UI/L)*	6959	7800	212 *
*EMG*	Myogenic involvement	Myogenic involvement	Negative
*Muscle MRI*	Multifocal edema	Multifocal edema	Multifocal edema
*Muscle biopsy*	Not performed	Positive	Not performed
Neuromuscular junction involvement	Probable	Probable	Yes, Lambert–Eaton
*Autoantibodies in serum*	Ab. anti-titin	Ab. anti-titin	Ab. anti-VGCC P/Q Ab. anti-GAD
*EMG*	No decrement	No decrement	Isolated axonal loss without increment
Other organ involvements	Multifocal myelitis Myocarditis		Possible axonal neuropathy (EMG) Pneumonitis
**Treatment**			
Corticosteroid therapy (grams of prednisone equivalent)	Yes (11.3 g)	Yes (7 g)	Yes (5.5 g)
Plasma exchange (*n*)	Yes (8)	No	No
Intravenous immunoglobulin (g/kg)	Yes (2 g/kg)	Yes (2 g/kg)	No
Immunosuppressants and modulators	Tofacitinib (18 days) Tacrolimus (maintained)	Abatacept (6 infusions) Ruxolitinib (40 days)	Infliximab (1 infusion)
Neurological treatments	Yes	No	Yes
*- Amifampridine*	Yes (6 days)		Yes (35 days)
*- Mestinon*	Yes (65 days)		Yes (5 days)
Outcome in ICU Long-term outcome	Favorable Alive, cancer stabilized	Favorable Died from pneumonitis	Death (tracheostomy obstruction)

ICI: immune checkpoint inhibitor; CPK: creatine phosphokinases, N < 195 IU/L). * CPKs were not assessed at admission for patient #3; the value given corresponds to the first assessment, 28 days after admission. High liver enzyme levels at admission suggest muscle involvement; EMG: electroneuromyogram; MIP: maximal inspiratory pressure, N > 75 cmH20; FVC: forced vital capacity.

## Data Availability

The data that support the findings of this study are available from the corresponding author upon reasonable request. The data are not publicly available due to privacy concerns.

## Supplementary Materials

The following supporting information can be downloaded at: https://www.mdpi.com/article/10.3390/reports7040084/s1, Supplementary S1: Diaphragmatic electroneuromyogram of patient #3.
