# Supplementary material for: Diaphragmatic Palsy Due to a Paraneoplastic Autoimmune Syndrome Revealed by Checkpoint Inhibitors
Source: Reports (MDPI). 2024 Oct 11;7(4):84. doi: 10.3390/reports7040084 (PMC12199867; doi:10.3390/reports7040084)

Supplementary S1: Diaphragmatic electroneuromyogram of patient #3

Phrenic conduction :

Stimulation at the neck. Recording at the 7th intercostal space.

Nerve	Lat. ms	Amp. 1-2 mV	Surface mVms
Left phrenic nerve - diaphragm	9,05	>0,1	0,3
Right phrenic nerve - diaphragm	10,20	>0,1	0,1

Search for increment :

Nerve / Site	Lat. ms	Amp. 1-2 mV	Surface mVms	Amp. 1-2 %	Surface %
Right Trapezius – spinal nerve					
Rest	2,80	1,9	13,5	100	100
After 30s of maximal effort	2,85	1,9	13,3	98,2	98,1

Search for decrement (3 Hz) :

Muscle	Ampl 1 mV	Dec A 1.5 %	Surf 1 mVms	Dec S 1,5 %	Déc. A9 %	Déc.s 9 %	Faci(amp) %
Right Trapezius – Spinal	2,6	-5	11,2	-7,4	-3	-6,9	100
Right orbicular - Facial	0,7	-0,2	1,6	-1,3	0,5	4,4	100

CONDUCTION MOTRICE G PHRENIQUE - DIAPHRAGME

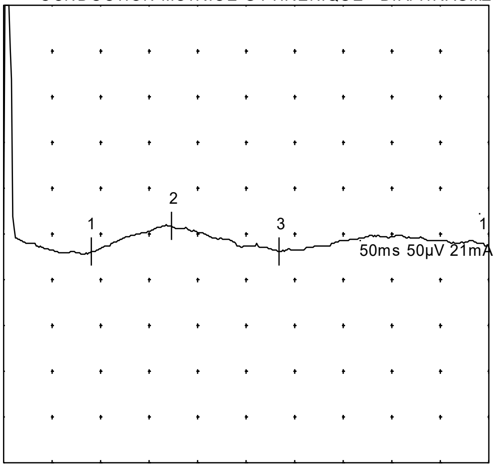

CONDUCTION MOTRICE D PHRENIQUE - DIAPHRAGME

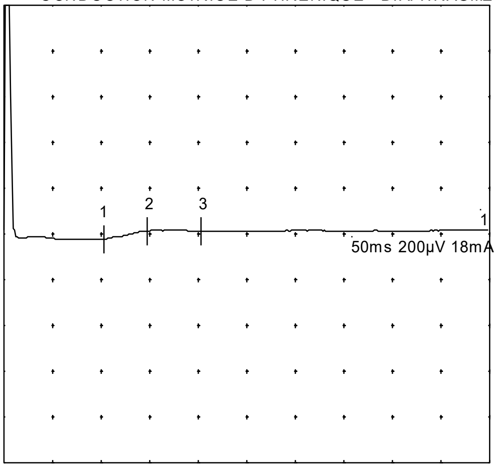

CONDUCTION MOTRICE D NERF

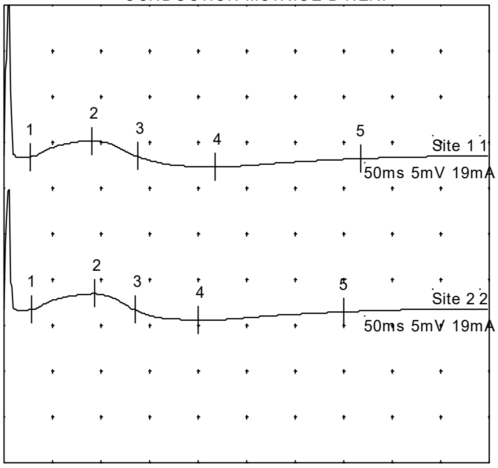

STIM REP D TRAPEZIUS (U) - SPINAL

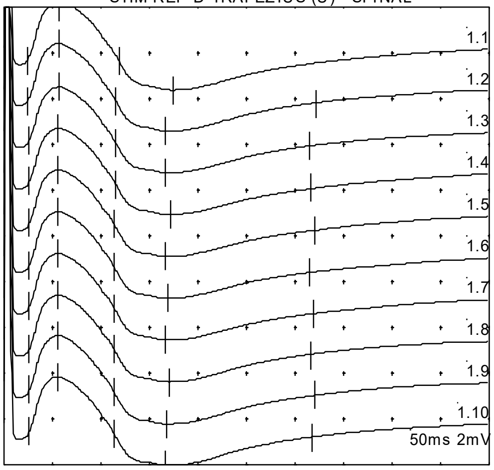

STIM REP D ORB OCULI - FACIAL

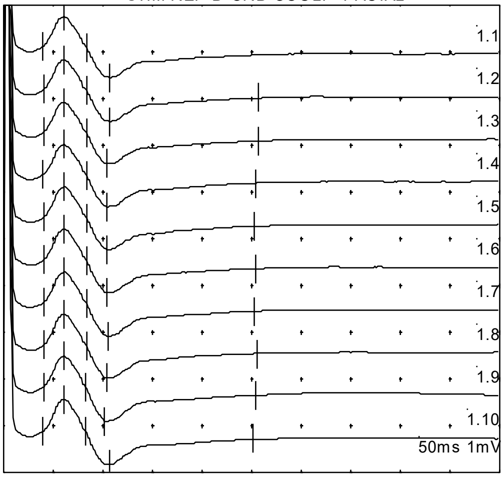

Supplement: Supplementary file 1 [file reports-07-00084-s001.zip › reports-3214001-supplementary.pdf]
